# AOP-Based Transformation of Abacavir in Different Environments: Evolution Profile of Descyclopropyl-Abacavir and In Silico Toxicity Assessment of the Main Transformation Products

**DOI:** 10.3390/molecules28041866

**Published:** 2023-02-16

**Authors:** Eleni Evgenidou, Konstantina Vasilopoulou, Lelouda-Athanasia Koronaiou, George Kyzas, Dimitrios Bikiaris, Dimitra Lambropoulou

**Affiliations:** 1 Laboratory of Environmental Pollution Control, Department of Chemistry, Aristotle University of Thessaloniki, 541 24 Thessaloniki, Greece; 2 Centre for Interdisciplinary Research and Innovation (CIRI-AUTH), Balkan Centre, 570 01 Thessaloniki, Greece; 3 Department of Chemistry, International Hellenic University, 654 04 Kavala, Greece; 4 Laboratory of Polymer Chemistry and Technology, Department of Chemistry, Aristotle University of Thessaloniki, 541 24 Thessaloniki, Greece

**Keywords:** abacavir, antiviral drug, AOPs, transformation products, ECOSAR, evolution profiles

## Abstract

This study explores the photocatalytic transformation of the antiviral drug abacavir employing different advanced oxidation processes (AOPs) such as UV/TiO_2_, UV/MOF/H_2_O_2_, UV/MOF/S_2_O_8_^2−^, UV/Fe^2+^/H_2_O_2_, and UV/Fe^2+^/S^2^O_8_^2−^. All processes appear to be effective in eliminating abacavir within a few minutes, while the evolution profile of the basic transformation product, descyclopropyl-abacavir (TP-247) was also monitored. Moreover, the implementation of the most efficient technologies towards the removal of abacavir in different matrices such as wastewater effluent and leachate was also assessed, revealing that the organic matter present or the inorganic constituents can retard the whole process. Four major transformation products were detected, and their time-evolution profiles were recorded in all studied matrices, revealing that different transformation pathways dominate in each matrix. Finally, the prediction of the toxicity of the major TPs employing ECOSAR software was conducted and showed that only hydroxylation can play a detoxification role in the treated solution.

## 1. Introduction

The presence of pharmaceutical compounds (PhCs) in the environment is an indisputable fact [1]. Their occurrence in surface, groundwater, and drinking water has been confirmed, posing various adverse effects since long-term exposure to these contaminants can provoke reproductive abnormalities, behavioral changes, mutations, cancer, or even mortality not only for wildlife populations but for humans as well [2]. PhCs can enter the wastewater inflows due to improper use, since overconsumption can lead to excretion of unmetabolized PhCs through urine and faces, but also due to proper use, considering that unused drugs are improperly discarded in the toilet, the drain, or into waste containers [1,3]. Their incomplete removal in the wastewater treatment plants benefits their intake into the water cycle, while a large portion of unused PhCs that end up in landfill sites for solid waste result in leachates, thus also posing a threat to groundwater [4].

Their increased detection in the various environmental compartments not only stems from the escalated consumption in the last few years due to the higher standards of living but also from the improvement of the analytical techniques applied. Consequently, not only overconsumed were PhCs identified in analytical applications in various aqueous matrices but also compounds with very low environmental levels (ng/L) that only recently did it become possible to address [5]. Among the various therapeutic classes of PhCs detected, antiviral drugs can be considered of utmost importance since they have been characterized to be hazardous with regard to their toxicity towards algae, daphnids, and fish [6]. Abacavir is an antiretroviral (ARV) drug used in the treatment of retroviral infections, primarily related to the human immunodeficiency virus type 1 (HIV-1). Despite its removal from WWTPs (>99%), abacavir has been detected in wastewaters, groundwater, surface waters, and even drinking water [7] in concentrations ranging from ng L^−1^ to μg L^−1^ [8]. 

Various techniques have been applied and evaluated to remove pharmaceutical compounds from water or wastewater [9]. Advanced oxidation processes (AOPs) have been well established to exhibit high removal efficiency for various classes of pharmaceuticals while offering flexible application [10]. Despite the diversity in the reagents and catalysts used in the AOPs, they are all characterized by a common feature: the production of hydroxyl radicals. The latter can attack unselectively all organic compounds, offering efficient degradation not only of the parent compound but also of their transformation products (TPs), and consequently, they can lead to high levels of mineralization and detoxification of the treated solutions [11,12]. Moreover, sulfate radical-based AOPs (SR-AOPs), a subcategory of traditional AOPs, are gaining popularity due to the high selectivity of the produced sulfate radicals towards specific contaminants, allowing for higher-than-ever removal efficiencies [13]. 

Previous studies exhibited very high removal yields for abacavir through biological treatment [7,8]. However, the rapid breakdown of the parent compound into its intermediates may yield misleading results concerning its complete removal, since various abacavir TPs were detected in the WWTP effluents, indicating the inadequacy of the biological treatment for complete mineralization.

Under this light, the main scope of this study was the application of different AOPs aiming not only for the degradation of abacavir but also the elimination of its TPs by employing different reacting systems such as UV/TiO_2_, UV/MOF/H_2_O_2_, UV/MOF/S_2_O_8_^2−^, UV/Fe^2+^/H_2_O_2_, and UV/Fe^2+^/S_2_O_8_^2−^. Moreover, the implementation of the most efficient technologies for the removal of abacavir in different matrices such as wastewater effluent and leachate was also assessed. Four major transformation products were detected, and their time-evolution profiles were recorded in order to obtain greater insight into the oxidation efficiency of each applied technology. Finally, the prediction of the toxicity of the major identified TPs employing ECOSAR software was conducted in order to facilitate and support a more integrated evaluation of each utilized AOP. To the best of our knowledge, the photocatalytic treatment of a specific antiviral drug with different AOPs has never been conducted before.

## 2. Results and Discussion

### 2.1. Preliminary Studies

Prior to the application of any AOP, photolytic experiments were conducted in order to assess any contribution from direct photolysis of the target compound. Abacavir appears to be photolabile due to the cyclopropyl moiety, but within a two-hour treatment, only 20% degradation was achieved. Additional experiments performed in the dark for possible hydrolysis revealed no degradation within this period, and consequently, any observed degradation can be solely attributed to the applied photochemical method.

### 2.2. Heterogenous AOPs

#### 2.2.1. TiO_2_-Photocatalysis

**Figure 1:**
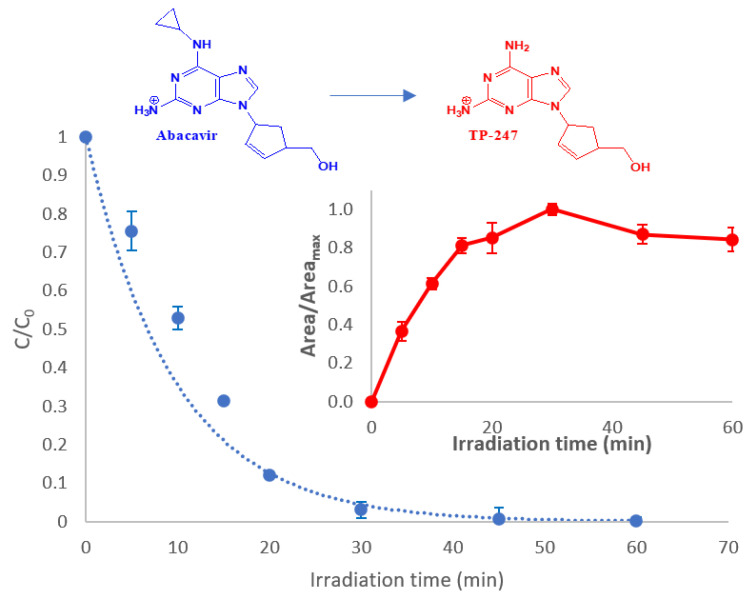
Degradation of abacavir. Inset: evolution profile of the most abundant TP (TP-247). C_0_ = 10 mg/L, CTiO_2_ = 10 mg/L, matrix = ultrapure water.

Photocatalytic treatment of abacavir with 10 mg/L TiO_2_ led to complete disappearance of the compound within 60 min, as presented in Figure 1. The degradation rate (k = 0.104 min^−1^) was calculated based on the fitting of the exponential curve described for first-order reactions (R^2^ > 98%, y = exp(−k t) where: t is the irradiation time and k is the rate constant). 

Among the transformation products that are produced during treatment (see Section 2.6), the most abundant intermediate that is formed is descyclopropyl abacavir, labeled as TP-247. The rapid and abundant transformation of the parent compound to TP-247 necessitated the monitoring of the latter in every applied AOP.

Consequently, in the inset of Figure 1, the evolution profile of TP-247 is presented. As can be observed, TP-247 reaches its maximum at 30 min, while at the same time point, more than 95% of abacavir is transformed. After this time interval, TP-247 concentration declines; however, no significant reduction is observed within 60 min of treatment, requiring more prolonged irradiation times for its degradation to be achieved.

**Figure 2:**
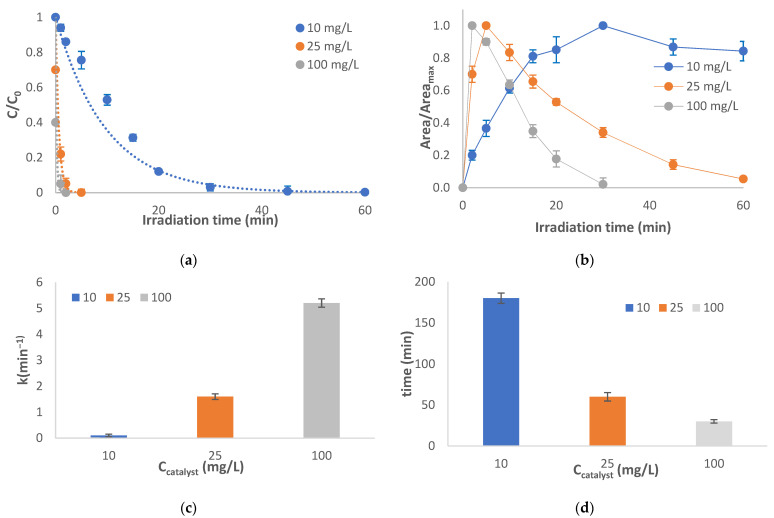
Photocatalytic treatment under different concentrations of catalyst: (**a**) degradation of the parent compound, (**b**) evolution profile of TP-247, (**c**) rate constant of the degradation of abacavir, (**d**) time required for complete disappearance of TP-247. C_0_ = 10 mg/L, C_TiO2_ =10, 25 or 100 mg/L, matrix = ultrapure water.

In order to study the effect of the amount of the catalyst, higher concentrations of TiO_2_ were employed. In Figure 2, the photocatalytic degradation of abacavir in the presence of 10, 25, and 100 mg/L is depicted, while the evolution profile of TP-247 is also presented.

Obviously, abacavir disappears completely within two or five min when the concentration of the catalyst is 100 mg/L or 25 mg/L, respectively, instead of the 60 min that are required at 10 mg/L of catalyst (Figure 2a). This noteworthy increase in reaction rate of the parent compound from 0.104 to 1.6 and finally to 5.204 min^−1^ (Figure 2c) can be attributed to the increase in the production of oxidative species and the available active sites on the catalyst’s surface [14]. Furthermore, the evolution of TP-247 is also accelerated (Figure 2b). At 100 mg/L of catalyst, it reaches its maximum concentration within 5 min, and only 30 min are required for its complete disappearance (compared to 30 min and 180 min, respectively, at 10 mg/L of catalyst (Figure 2d).

#### 2.2.2. MOF Based Photocatalysis

**Figure 3:**
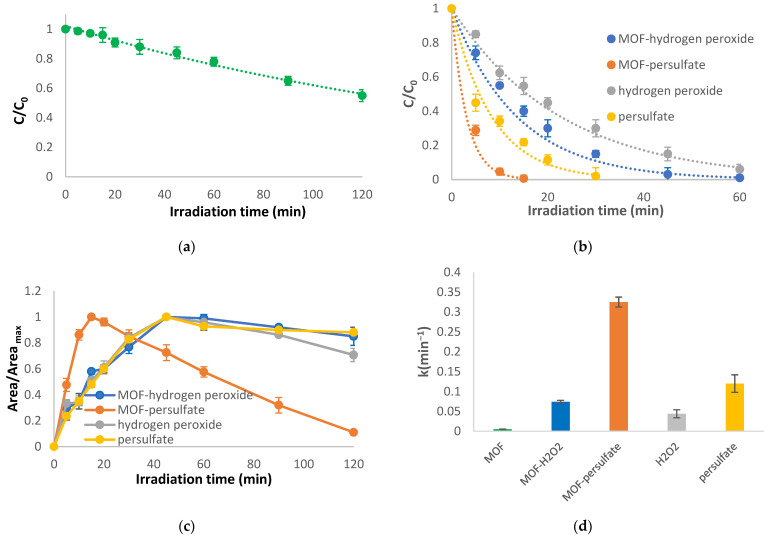
Photocatalytic treatment using MOF-based AOPs: (**a**) degradation of the parent compound using the UV/MOF process, (**b**) degradation of the parent compound using the UV/MOF/H_2_O_2_, UV/MOF/S_2_O_8_^2−^, UV/H2O2, and UV/S_2_O_8_^2−^ processes, (**c**) evolution of TP-247 using the UV/MOF/H_2_O_2_, UV/MOF/S_2_O_8_^2−^, UV/H2O2, and UV/S_2_O_8_^2−^ processes, (**d**) rate constants of the degradation of abacavir. C_0_ = 10 mg/L, C_MOF_ = 100 mg/L, C_oxidant_ = 15 mg/L, matrix = ultrapure water.

Photocatalytic treatment of abacavir in the presence of 100 mg/L MOF catalyst (Basolite F-300) did not achieve high degradation efficiencies as presented in Figure 3a (k = 0.005 min^−1^). As reported elsewhere, fast recombination of the generated electron-hole pairs, reducing photocatalytic efficiency, is responsible for the limited degradation of the target compound [15]. However, the addition of oxidants (hydrogen peroxide or persulfate, Figure 3b) can enhance the photocatalytic efficiency of MOFs since they prohibit the recombination of the electron-hole pairs and can produce additional oxidative radicals either through self-activation by irradiation or through Fenton type reactions when iron-based MOFs are used [15,16]. For comparison, UV/H_2_O_2_ and UV/S_2_O_8_^2−^ processes without the addition of the catalyst were also applied. Although fast degradation is achieved with the presence of oxidants without a catalyst (k = 0.044 and 0.12 min^−1^ for UV/H_2_O_2_ and UV/S_2_O_8_^2−^, respectively), the addition of the MOF catalyst promotes further degradation efficiency of the parent compound, thus achieving higher reaction rates (k = 0.074 and 0.325 min^−1^ for UV/MOF/H_2_O_2_ and UV/MOF/S_2_O_8_^2−^, respectively, Figure 3d). The same profile is also observed for the formation of TP-247. Higher degradation rates were accomplished with the UV/MOF/S_2_O_8_^2−^ process, and the specific TP acquires its maximum at 10 min (instead of the 45 min that are required in the other processes) and it is almost completely degraded within 120 min.

### 2.3. Homogenous AOPs

#### 2.3.1. Photo-Fenton Reaction 

**Figure 4:**
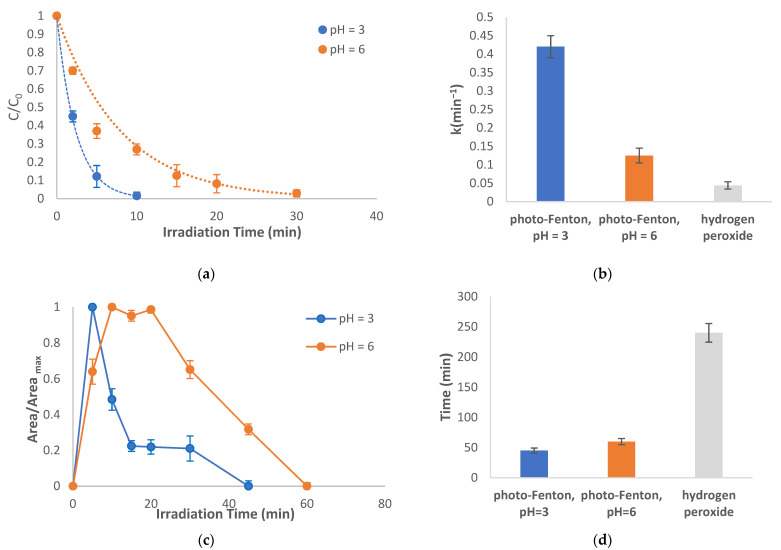
Photocatalytic treatment using the photo-Fenton reaction: (**a**) degradation of the parent compound at pH = 3 and 6, (**b**) rate constants of the degradation of abacavir, (**c**) evolution profiles of TP-247, and (**d**) time required for complete disappearance of TP-247. C_0_ = 10 mg/L, CFe^2+^ =1 mg/L, C_oxidant_ = 15 mg/L, matrix = ultrapure water.

Photocatalytic treatment of abacavir with the photo-Fenton reaction (UV/Fe^2+^/H_2_O_2_) leads to rapid degradation of the parent compound within 10 min (Figure 4a). The reaction was conducted at two different pH values (3 and 6) confirming once more the need for acidification of the solution [17]. At pH = 6, much slower reaction rates were achieved however, both photo-Fenton processes are decidedly faster than illuminated hydrogen peroxide alone (added for comparison reasons) (k = 0.42, 0.125, and 0.044 min^−1^ for UV/Fe^2+^/H_2_O_2_, at pH = 3, UV/Fe^2+^/H_2_O_2_, at pH = 6, and UV/H_2_O_2_, respectively) (Figure 4b). The same profile was also observed for TP-247 (Figure 4c,d).

#### 2.3.2. Photo-Fenton-like Reaction

**Figure 5:**
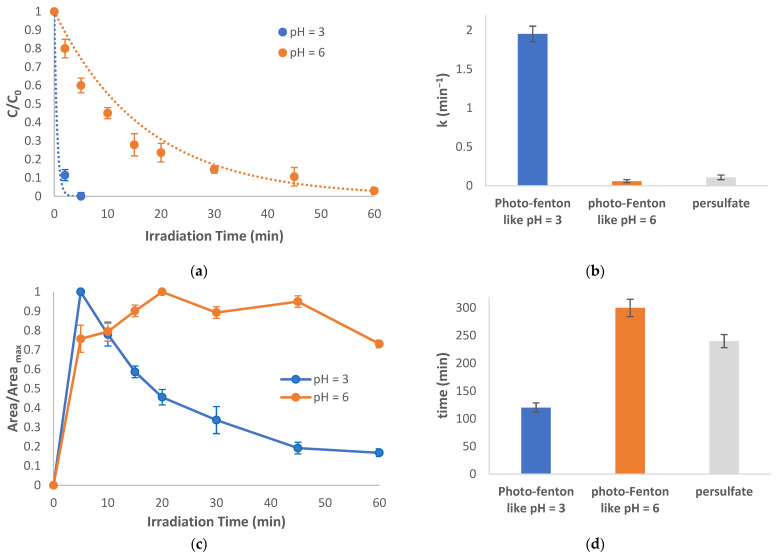
Photocatalytic treatment using a photo-Fenton-like reaction: (**a**) degradation of the parent compound at pH = 3 and 6, (**b**) rate constants of the degradation of abacavir, (**c**) evolution of TP-247, and (**d**) time required for complete disappearance of TP-247. C_0_ = 10 mg/L, CFe^2+^ =1 mg/L, C_oxidant_ = 15 mg/L, matrix = ultrapure water.

Accordingly, the photocatalytic treatment of abacavir with the photo-Fenton-like reaction (Fe^2+^/S_2_O_8_^2−^) leads to even more rapid degradation of the parent compound within 5 min (Figure 5a). The reaction was also conducted at two different pH values (3 and 6), with 60 min of treatment required for complete degradation at a pH of 6. Comparison with illuminated persulfate reveals that the photo-Fenton-like process proceeds at higher reaction rates (k = 1.953, 0.059, and 0.108 min^−1^ for UV/Fe^2+^/S_2_O_8_^2−^ at pH = 3, UV/Fe^2+^/S_2_O_8_^2−^_,_ at pH = 6, and UV/S_2_O_8_^2−^ respectively); however, pH = 6 appears to have a detrimental effect since slower reaction rates were achieved compared with the other applied processes. (Figure 5b). The same tendency was also observed for TP-247 where the UV/Fe^2+^/S_2_O_8_^2−^ at pH = 3, appears to be the process with the higher reaction rate (Figure 5c,d).

A comparison of all the applied processes reveals the higher efficiency of the heterogenous photocatalysis with 100 mg/L of catalyst (TiO_2_). Comparing the two oxidants used, it can be observed that persulfate addition demonstrates higher degradation efficiencies as well as higher reaction rates in photo-Fenton type reactions and in MOF based photocatalysis. This is in agreement with previous studies exhibiting that higher radical yields are produced when persulfate is employed instead of hydrogen peroxide due to the lower bond energy of the peroxide bond O-O in persulfate compared to hydrogen peroxide [18]. Moreover, it may be concluded that sulfate radicals can be more reactive towards abacavir degradation compared to hydroxyl radicals. This is probably attributed to the mechanism by which sulfate radicals usually react with organic compounds. Based on previous bibliography, sulfate radicals are more selective, and they usually react through an electron transfer mechanism [19]. Moreover, Prasse et al. have previously demonstrated that the main site of reaction during photodegradation of abacavir is the cyclopropyl ring, initiated by a one-electron oxidation of the cyclopropylamine moiety, leading to the formation of a cyclopropylaminium radical cation, which in turn takes part in subsequent reactions resulting in the formation of various products [20]. Obviously, this initiation is more favored in the presence of sulfate radicals in contrast with hydroxyl radicals, which tend to react with other mechanisms such as the electrophilic addition to π bonds or H abstraction [19].

### 2.4. Effect of Scavengers

In order to examine the effects of various reactive species that take place in the three most efficient types of AOPs, different scavengers have been employed at fixed concentrations. More specifically, potassium iodide (KI) for holes, isopropanol (C_3_H_8_O) for hydroxyl and sulfate radicals, and sodium azide (NaN_3_) for scavenging singlet oxygen [21]. Based on the results presented in Table S1, it is obvious that different reactive species play the dominant role in each applied AOP. Consequently, for heterogenous photocatalysis, hydroxyl radicals, sulfate radicals, and holes appear to be the dominant oxidative species. However, small differences are spotted between TiO_2_ or MOF-based photocatalysis since in the former, a larger inhibitory effect is provoked by the addition of isopropanol, exhibiting that hydroxyl radicals play the dominant role in the abacavir oxidation mechanism, whereas potassium iodide causes a greater inhibitory effect in the latter, revealing the dominant role of the holes. Besides, previous studies have already demonstrated that the addition of oxidants in MOF-based photocatalysis promotes the oxidation process by preventing the recombination of the electron-hole pairs and allowing holes to be the major contributors in the oxidation mechanism [15,22]. In homogenous photocatalysis, a very strong inhibition was caused by the addition of isopropanol, while in all three types of AOP, a lower inhibition was observed with the addition of azide, indicating the smaller contribution of singlet oxygen. 

### 2.5. Effects of the Matrix

**Figure 6:**
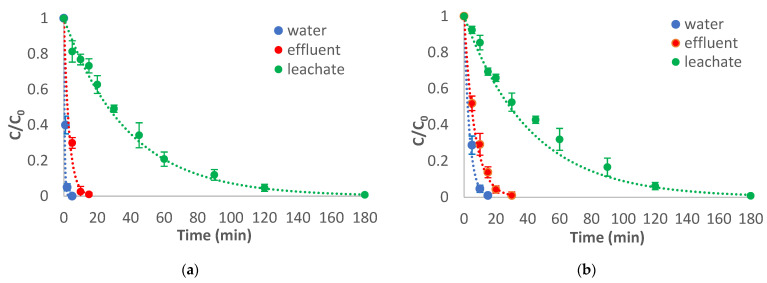

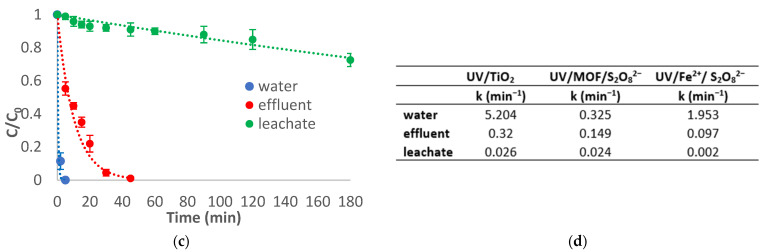
Photocatalytic degradation of abacavir in different matrices using (**a**) UV/TiO_2_ photocatalysis, (**b**) UV/MOF/S_2_O_8_^2−^ (**c**) UV/Fe^2+^/S_2_O_8_^2−^, and (**d**) calculated k values for all applied AOPs in different matrices. C_0_ = 10 mg/L, C_catalyst_ = 100 mg/L, CFe^2+^ = 1 mg/L, C_oxidant_ = 15 mg/L.

In order to study the effect of matrix constituents on the degradation kinetics, the three types of applied AOPs—heterogenous photocatalysis with TiO; heterogenous photocatalysis with MOF/oxidant; and homogenous photocatalysis—were tested in different matrices with high and low pollutant loads, such as leachate and wastewater effluent, respectively. Moreover, since higher efficiencies were achieved when persulfate was applied as an oxidant, the UV/ MOF/S_2_O_8_^2−^ and UV/ Fe^2+^/S_2_O_8_^2−^ at pH = 3 were selected to be tested instead of H_2_O_2_. In Figure 6, the photocatalytic degradation of abacavir in different matrices using different AOPs is depicted. 

Obviously, photocatalytic degradation of abacavir proceeds more rapidly in water compared to the other matrices for all applied AOPs. The presence of organic matter appears to be the major retarding factor since an increase in COD causes a noticeable decrease in the degradation rate of the target compound by consuming the produced radicals and other oxidative species. Moreover, inorganic ions can also be a retarding factor since they can act as radical scavengers, forming less reactive species, or they can occupy the active sites on the surface of a photocatalyst (in the case of heterogeneous photocatalysis), thus reducing its activity [23]. Finally, light attenuation due to inner filtering by colored dissolved substances, especially in the case of leachate, can also play an Inhibiting role in the applied processes [24].

### 2.6. Identification of TPs

**Figure 7:**
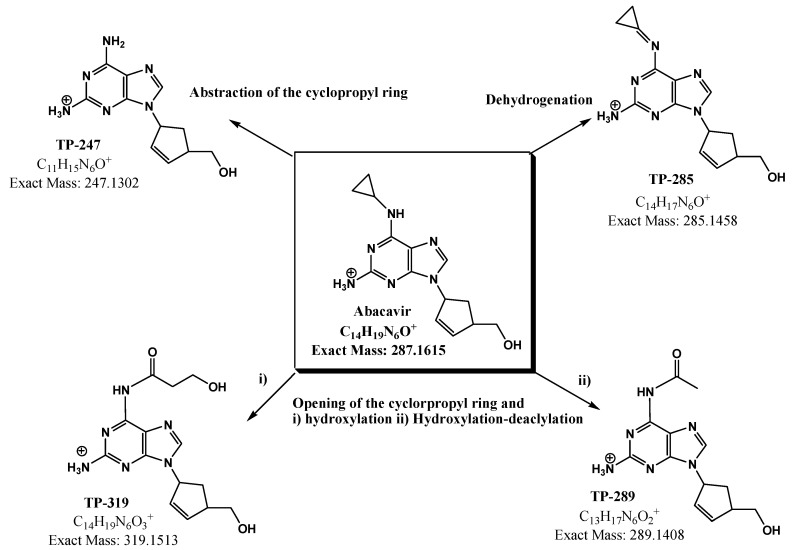
Main transformation products identified during photocatalytic treatment of abacavir.

As previously reported, the main site of reaction during photolytic degradation of abacavir is the cyclopropyl ring [20]. Four major transformation products (TPs) were identified during the photocatalytic degradation of abacavir, with all the applied AOPs (Table 1) arising from three major transformation routes: (i) detachment of the cyclopropyl ring, (ii) opening of the cyclopropyl ring and subsequent hydroxylation/oxidation/dealcylation, and finally (iii) dehydrogenation (Figure 7).

The most abundant TP, TP-247, arises from the scission of the C-N bond and the subsequent abstraction of the cyclopropyl moiety. On the other hand, the oxidative radicals formed in the applied AOPs can attack the cyclopropyl ring, leading to its opening and finally to its hydroxylation, forming TP-319. Continuous attack can further lead to the formation of an acetamide, the dealkylated product labeled as TP-289. Finally, dehydrogenation led to the formation of the cyclopropylimine derivative labelled as TP-285.

In order to obtain more integrated knowledge concerning the outcome of each applied photocatalytic process, the in silico ecotoxicity predictions of the identified TPs were calculated by applying the ECOSAR software. The parent compound and TP structures were inserted in the form of SMILES, whereas the results were divided according to the aquatic organism (*fish*, *daphnids*, *algae*), and they are expressed as mg/L. The chemical class with the lowest estimated values (worst case scenario) was selected for further assessment. The calculated values of toxicity were then characterized as “very toxic” if they ranged from 0.0 to 1.0, as “toxic” from 1 to 10, as “harmful” from 10 to 100, and as “non-toxic” if the values were above 100. The results are presented in Table 2.

Obviously, abacavir appears to be harmful to *fish* and toxic or very toxic for *daphnids* and *green algae*, respectively, in terms of acute toxicity, while being very toxic for all trophic levels in terms of chronic toxicity. Transformation of the parent compound does not appear to affect the toxicity since all TPs retain the toxic character of abacavir. However, hydroxylation appears to play a detoxification role since TP-319 presents the lowest toxicity in all trophic levels and in both types of toxicity (acute and chronic).

### 2.7. Evolution of TPs in Different Matrices

**Figure 8:**
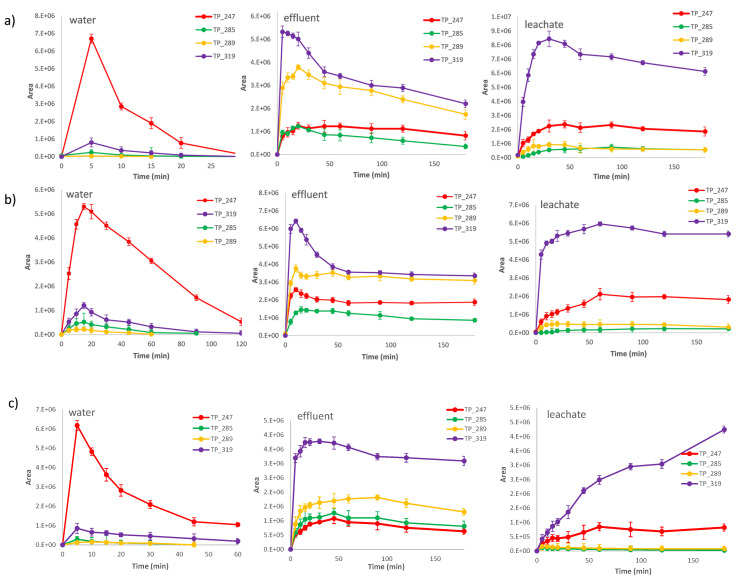
Evolution profile of the basic TPs of abacavir in three different matrices during heterogenous and homogeneous photocatalysis (**a**) UV/TiO_2_, (**b**) UV/MOF/S_2_O_8_^2−^, and (**c**) UV/Fe^2+^/S_2_O_8_^2−^ C_0_ = 10 mg/L, C_catalyst_ = 100 mg/L, CFe^2+^ =1 mg/L, C_oxidant_ = 15 mg/L.

The main identified TPs of abacavir were monitored during all types of AOPs and in different matrices. In Figure 8, the evolution profiles of the main degradation TPs during heterogeneous photocatalysis (Figure 8a: UV/TiO_2_ and Figure 8b: UV/MOF/S_2_O_8_^2−^) and during homogeneous photocatalysis (Figure 8c: UV/Fe^2+^/S_2_O_8_^2−^**)** in three different matrices (water, effluent, and leachate) are presented. 

As depicted, higher reaction rates are observed in ultra-pure water for all applied AOPs, as already observed for the parent compound in Figure 6. The organic load in the effluents and the even higher load in the leachate cause a progressive retardation to the evolution profiles of all TPs (a plateau) since the produced reactive species can be consumed by the former. Probably, higher quantities of catalysts or oxidants and more prolonged irradiation times are required for the TPs to be degraded in such matrices. Moreover, another observation is that the basic transformation pathway changes depending on the matrix. Consequently, TP-247 is the major TP when the treatment is conducted in water, but in the other two matrices, TP-319 appears to be the dominant one for all applied AOPs. Consequently, in the two matrices (effluent and leachate), other inorganic constituents such as chloride, bicarbonates, or nitrate ions are present. These constituents can act as radical scavengers, producing other secondary radicals that are less reactive than the primary sulfate or hydroxyl radicals they produce. These secondary radicals can also oxidize the target compound, but at lower rates [25,26]. Moreover, the high concentration of organic matter dramatically reduced the available primary radicals for the oxidation of the target compound. The combination of these two factors leads to milder oxidative conditions in the system, thus permitting the opening of the cyclopropyl ring with its subsequent hydroxylation instead of the rapid abstraction. Consequently, milder oxidative conditions can promote the detoxification of the solution since the hydroxylated TP-319 is the less toxic by-product; however, prolonged irradiation times are required for complete degradation to be achieved. 

## 3. Materials and Methods

### 3.1. Reagents 

Abacavir (purity ≤ 100%) was purchased from Sigma Aldrich (Darmstadt, Germany). Stock solutions were prepared in ultrapure water and stored at −20 °C. Working solutions of 100 mL volume were prepared daily prior to treatment, employing 10 mg L^−1^ as a starting concentration. Since synthesis of new photocatalytic materials was beyond the scope of the present study, only commercially available catalysts were used and compared. Consequently, the photocatalysts employed in the experiments were Titanium dioxide P25 from Evonik (Darmstadt, Germany) (particle size 20–30 nm; crystal structure: ~80% anatase and 20% rutile; surface area: 56 m^2^ g^−1^; zero point of charge ≈ 6.3–6.8) and Basolite F300 (Fe-BTC, Iron 1,3,5-benzenetricarboxylate; CAS Number:1195763-37-1, produced by BASF, BET; surface area: 1300–1600 m^2^/g; bulk density: 0.16 0.35 g/cm^3^) from Sigma Aldrich/Merck (Darmstadt, Germany). The oxidants hydrogen peroxide (H_2_O_2_, 30% *w*/*v*) and potassium persulfate (K_2_S_2_O_8_, 99+%) were obtained from Panreac (Barcelona, Spain) and Chem-LabNV (Zedelgem, Belgium), respectively. For the analysis, LC-MS-grade methanol was purchased from Merck (Darmstadt, Germany), while formic acid (HCOOH) was obtained from Sigma Aldrich (Darmstadt, Germany). The leachate sample was collected from a landfill site in northern Greece in amber glass vessels and stored at 4 °C to prevent further biological degradation of the sample. The initial physicochemical characteristics were: pH = 7.8; total solids = 19825 mg L^−1^; COD = 5936 mg L^−1^; BOD = 98 mg L^−1^; NH^4+^-N = 584 mg L^−1^; NO^3—^N = 231 mg L^−1^; NO^2—^N = 1102 mg L^−1^. The wastewater effluent was from an urban wastewater treatment plant (WWTP) with the following characteristics: BOD = 19.3 mg L^−1^; COD = 62.4 mg L^−1^; SS = 10.3 mg L^−1^; and pH = 7.9.

### 3.2. Photolysis and Photocatalysis Experiments 

All photolytic and photocatalytic experiments were carried out in duplicate using the Atlas Suntest CPS+ (Linsengericht Germany) photoreactor equipped with a xenon lamp (1500 W) at a light intensity of 500 W m^−2^ and an air-cooler and cut-off filter (<290 nm Figure S1). Working solutions were added to the pyrex reactor and mixed using magnetic stirring. In the case of heterogeneous photocatalysis, the photocatalyst was added prior to illumination, and an equilibration time of 30 min in dark conditions was applied to estimate any adsorption of the studied pharmaceutical on the catalyst’s surface. For the photo-Fenton process, the pH was initially adjusted to pH = 3. After the addition of the oxidants, samples were irradiated and withdrawn from the reactor at specific time intervals, centrifuged, filtered, and then proceeded for analysis.

### 3.3. Analytical Processes

#### Low and High-Resolution Mass Spectrometry

For the determination of Abacavir (Abc) and its major TP (descyclopropyl abacavir labeled as TP-247), an LC-MS system consisted of a SIL 20A autosampler and a LC-20AB pump from Shimadzu (Kyoto, Japan). Detection was carried out using an SPD 20A DAD detector coupled in series with the LC-MS 2010EV mass selective detector, equipped with an atmospheric pressure electrospray ionization source (ESI), running in the positive ionization mode. The analytical column used was a C18, 150 × 4.6 mm with a 3.5 µm particle size (Pathfinder) at 40 °C. LC-MS grade water-0.1% formic acid (solvent A) and methanol (solvent B) at a flow rate of 0.4 mL min^−1^ were used as mobile phases. The gradient elution program started with 70% A, kept constant for 1 min, changed to 10% A at 6 min, and returned to initial conditions at 7 min. The total run time was 20 min. Analyte detection was based on the precursor molecular ion [M+H]+ (*m*/*z* = 287 and 247 for abacavir and TP-247, respectively) in the selected-ion monitoring mode (SIM). The LOD and LOQ values were found to be 35 and 100 ng/mL, respectively.

The basic TPs arising from the photocatalytic degradation of abacavir were further characterized by an LC–Q Exactive Focus Orbitrap high-resolution mass spectrometry system equipped with a heated electro-spray ionization (HESI-II) probe in the positive ionization mode. Separation was achieved on a Thermo Hypersil GOLD C18 aQ column (50 × 2.1 mm, 1.9 μm), and the mobile phase consisted of (A) water LC-MS (0.1% formic acid) and (B) methanol (0.1% formic acid). The chromatographic gradient program was carried out within 15 min under the following conditions. 0 min, 10% B; 1.5 min, 10% B; 4 min, 60% B; 8 min, 70% B; 11–12 min, 100% B; 13 min, 10% B; 15 min, 10% B. The mass range was set at 50–1000 *m*/*z*, and the resolution was set at 70,000 and 17,500 (FWHM) in full-scan and data-dependent (ddMS2) discovery modes, respectively. Stepped collision energies (20, 30, and 45 eV) were used for fragmentation. The Xcalibur 4.1 software (Thermo Fisher Scientific Inc., Waltham, MA, USA) was used for instrument control and the elucidation of the structure of the formed TPs.

For the characterization of the leachate and wastewater, COD, NH^4+^-N, NO^3—^N, and NO^2−^-N measurements were carried out using a water/wastewater multiparameter photometer (Hanna), while BOD was measured by the respirometric method, using the OXI Top WTW measuring kit. COD was measured based on the EPA-approved dichromate method, NH^4+^-N on the Nessler method, NO^3−^-N on the cadmium reduction method, and NO^2−^-N on the chromotropic acid method. PH was measured using a Knick pH-meter. TSS were measured based on the EPA 2540 method.

## 4. Conclusions

Complete degradation of the antiviral drug abacavir has been achieved with all the applied processes. In heterogenous photocatalysis with TiO_2_, fast degradation has been observed within a few minutes. An increase in the concentration of the catalyst caused an increase in the reaction rate as well. Employing MOF as a catalyst caused a very slow degradation, while the addition of oxidants (H_2_O_2_ or S_2_O_8_^2−^) enhanced the degradation process. Photo-Fenton or photo-Fenton-like treatments were also efficient processes for degrading the target compound within a few minutes, while pH plays a dominant role in the specific processes. Comparison between the processes revealed the supremacy of heterogenous photocatalysis with TiO_2_ since it achieves higher reaction rates for abacavir and its main TP (TP-247) within 30 min. Among the other processes, only the photo-Fenton reaction could achieve complete disappearance of the main TP in less than 60 min of treatment time. Application of the most competent of the studied processes in other matrices showed that prolonged irradiation times are required for complete disappearance of the studied compound since the high load of organic matter or the inorganic species present could scavenge the oxidative radicals. Moreover, four main TPs were identified, resulting from cyclopropyl ring cleavage (TP-247), opening of the cyclopropyl ring and subsequent hydroxylation (TP-319), deacylation (TP-289), and dehydrogenation (TP-285), all of which appear to retain toxic properties as the parent compound according to the ECOSAR software. However, only hydroxylation can play a detoxification role since TP-319 exhibits the lowest toxic properties. Monitoring the evolution profiles of the identified TPs establishes the supremacy of heterogenous photocatalysis with TiO_2_ since it can accomplish their complete degradation within 30 min of treatment when applied in water. When the organic load increases (in wastewater effluent or leachate), the evolution profile proceeds at a much slower rate, thus requiring longer treatment times. Moreover, depending on the water matrix, different transformation pathways dominate in the treated solution since milder oxidative conditions are present. As a result, a comparison of all applied AOPs reveals that heterogenous photocatalysis appears to be a more efficient technique, even at high loaded matrices, for the removal of the target compound or the formed TPs. Moreover, employing TiO_2_ as a catalyst can give even more satisfying results since it achieves higher reaction rates with no extra addition of oxidants (compared to MOF catalysts) or pH adjustments required (compared to homogenous techniques). However, when applied on a large scale, the post-separation step for the removal of the catalyst might be a serious drawback to be considered.

## Figures and Tables

**Table 1:** LC–HRMS for the major TPs of abacavir.

TP Name/Fragments	tR (min)	Elemental Composition	*m*/*z* (theor)	*m*/*z* (exper)	RDBE	Δ (ppm)
**Abacavir**	**5.88**	**[C_14_H_19_N_6_O]^+^**	**287.1615**	**287.1608**	**8.5**	**−2.560**
*Fragment 1*		*[C_8_H_11_N_6_]^+^*	*191.1040*	*191.1036*	*6.5*	*−2.360*
*Fragment 2*		*[C_8_H_8_N_5_]^+^*	*174.0774*	*174.0771*	*7.5*	*−1.734*
*Fragment 3*		*[C_5_H_7_N_6_]^+^*	*151.0727*	*151.0724*	*5.5*	*−1.859*
*Fragment 4*		*[C_5_H_4_N_5_]^+^*	*134.0461*	*134.0459*	*6.5*	*−1.878*
*Fragment 5*		*[C_4_H_5_N_4_]^+^*	*109.0509*	*109.0511*	*4.5*	*1.259*
*Fragment 6*		*[C_4_H_3_N_4_]^+^*	*107.0352*	*107.0354*	*5.5*	*−1.657*
*Fragment 7*		*[C_6_H_7_]^+^*	*79.0542*	*79.0548*	*3.5*	*6.745*
**TP247**	**4.79**	**[C_11_H_15_N_6_O]^+^**	**247.1302**	**247.1296**	**7.5**	**−2.340**
*Fragment 1*		*[C_5_H_7_N_6_]^+^*	*151.0727*	*151.0724*	*5.5*	*−1.859*
*Fragment 2*		*[C_5_H_4_N_5_]^+^*	*134.0461*	*134.0459*	*6.5*	*−1.878*
*Fragment 3*		*[C_4_H_5_N_4_]^+^*	*109.0509*	*109.0511*	*4.5*	*1.259*
*Fragment 4*		*[C_4_H_3_N_4_]^+^*	*107.0352*	*107.0354*	*5.5*	*−1.657*
*Fragment 5*		*[C_4_H_2_N_3_]^+^*	*92.0243*	*92.0247*	*5.5*	*4.700*
*Fragment 6*		*[C_6_H_7_]^+^*	*79.0542*	*79.0548*	*3.5*	*6.745*
**TP319**	**4.99**	**[C_14_H_19_N_6_O_3_]^+^**	**319.1513**	**319.1504**	**8.5**	**−2.460**
*Fragment 1*		*[C_8_H_11_N_6_O_2_]^+^*	*223.0938*	*223.09329*	*6.5*	*−2.287*
*Fragment 2*		*[C_8_H9N_6_O]^+^*	*205.0832*	*205.0829*	*7.5*	*−1.782*
*Fragment 3*		*[C_5_H_6_N_5_O]^+^*	*152.0567*	*152.0561*	*5.5*	*0.879*
*Fragment 4*		*[C_5_H_7_N_6_]^+^*	*151.0727*	*151.0724*	*5.5*	*−1.859*
*Fragment 5*		*[C_5_H_4_N_5_]^+^*	*134.0461*	*134.0459*	*6.5*	*−1.878*
*Fragment 6*		*[C_4_H_5_N_4_]^+^*	*109.0509*	*109.0511*	*4.5*	*1.259*
*Fragment 7*		*[C_4_H_3_N_4_]^+^*	*107.0352*	*107.0354*	*5.5*	*−1.657*
*Fragment 8*		*[C_6_H_7_]^+^*	*79.0542*	*79.0548*	*3.5*	*6.745*
**TP289**	**5.30**	**[C_13_H_17_N_6_O_2_]^+^**	**289.1408**	**289.1399**	**8.5**	**−2.680**
*Fragment 1*		*[C_7_H_9_N_6_O]^+^*	*193.0832*	*193.0827*	*6.5*	*−2.204*
*Fragment 2*		*[C_5_H_6_N_5_O]^+^*	*152.0567*	*152.0561*	*5.5*	*0.879*
*Fragment 3*		*[C_5_H_7_N_6_]^+^*	*151.0727*	*151.0724*	*5.5*	*−1.859*
*Fragment 4*		*[C_5_H_4_N_5_]^+^*	*134.0461*	*134.0459*	*6.5*	*−1.878*
*Fragment 5*		*[C_4_H_5_N_4_]^+^*	*109.0509*	*109.0511*	*4.5*	*1.259*
*Fragment 6*		*[C_4_H_3_N_4_]^+^*	*107.0352*	*107.0354*	*5.5*	*−1.657*
*Fragment 7*		*[C_6_H_7_]^+^*	*79.0542*	*79.0548*	*3.5*	*6.745*
**TP285**	**5.82**	**[C_14_H_17_N_6_O]^+^**	**285.1458**	**285.1453**	**9.5**	**−1.820**
*Fragment 1*		*[C_8_H_9_N_6_]^+^*	*189.0883*	*189.0879*	*7.5*	*−2.279*
*Fragment 2*		*[C_8_H_6_N_5_]^+^*	*172.0618*	*172.0616*	*8.5*	*−1.173*
*Fragment 3*		*[C_7_H_8_N_5_]^+^*	*162.0774*	*162.0771*	*6.5*	*−1.801*
*Fragment 4*		*[C_7_H_9_N_4_]^+^*	*149.0822*	*149.0819*	*5.5*	*−1.495*
*Fragment 5*		*[C_4_H_5_N_4_]^+^*	*109.0509*	*109.0511*	*4.5*	*1.259*
*Fragment 6*		*[C_5_H_5_N_3_]^+^*	*107.0478*	*107.0479*	*5*	*1.506*
*Fragment 7*		*[C_6_H_7_]^+^*	*79.0542*	*79.0548*	*3.5*	*6.745*

**Table 2:** ECOSAR results for the acute/ chronic toxicity of Abacavir and its major TPs.

Compound Name	ECOSAR Classification	Acute Toxicity (mg/L) (LC_50_/EC_50_)			Chronic Toxicity (mg/L) (ChV)		
		*Fish*	*Daphnid*	*Green Algae*	*Fish*	*Daphnid*	*Green Algae*
**Abacavir**	Imidazoles	**20.85**	**4.93**	**0.49**	**0.16**	**0.13**	**0.49**
**TP-247**	Imidazoles	**119.23**	**13.62**	**0.91**	**0.55**	**0.38**	**1.03**
**TP-319**	Imidazoles	**800.57**	**60.46**	**3.25**	**2.77**	**1.72**	**3.98**
**TP-289**	Imidazoles	**183.92**	**18.89**	**1.20**	**0.78**	**0.53**	**1.38**
**TP-285**	Imidazoles	**36.30**	**6.92**	**0.61**	**0.24**	**0.19**	**0.63**

Ranges (mg/L) and their respective characterization: LC/EC_50_ ≤ 1, very toxic (red); 1 < LC/EC_50_ ≤ 10, toxic (orange); 10 < LC/EC_50_ ≤ 100, Harmful (yellow); LC/EC_50_ > 100, Not harmful (green).

## Data Availability

Not applicable.

## Supplementary Materials

The following supporting information can be downloaded at: https://www.mdpi.com/article/10.3390/molecules28041866/s1, Figure S1. Emission spectrum of the lamp used at the Atlas Suntest CPS+ (Germany) photoreactor. Table S1. Photocatalytic rate constants (k) and the inhibition percentage Δk% based on the addition of scavengers in the three optimum AOPs.
