# Supplementary material for: AOP-Based Transformation of Abacavir in Different Environments: Evolution Profile of Descyclopropyl-Abacavir and In Silico Toxicity Assessment of the Main Transformation Products
Source: Molecules. 2023 Feb 16;28(4):1866. doi: 10.3390/molecules28041866 (PMC9963360; doi:10.3390/molecules28041866)
Supplement: Supplementary file 1 [file molecules-28-01866-s001.zip › molecules-2183514-supplementary.pdf]

AOP-Based Transformation of Abacavir in Different Environments: Evolution Profile of Descyclopropyl-Abacavir and In Silico Toxicity Assessment of the Main Transformation Products

Eleni Evgenidou [1,2], Konstantina Vasilopoulou [1], Lelouda-Athanasia Koronaiou [1,2], George Kyzas [3], Dimitrios Bikiaris [4] and Dimitra Lambropoulou [1,2,*]

[1] Laboratory of Environmental Pollution Control, Department of Chemistry, Aristotle University of Thessaloniki, 541 24 Thessaloniki, Greece
[2] Centre for Interdisciplinary Research and Innovation (CIRI-AUTH), Balkan Centre, 570 01 Thessaloniki, Greece
[3] Department of Chemistry, International Hellenic University, 654 04 Kavala, Greece
[4] Laboratory of Polymer Chemistry and Technology, Department of Chemistry, Aristotle University of Thessaloniki, 541 24 Thessaloniki, Greece
* Correspondence: dlambro@chem.auth.gr

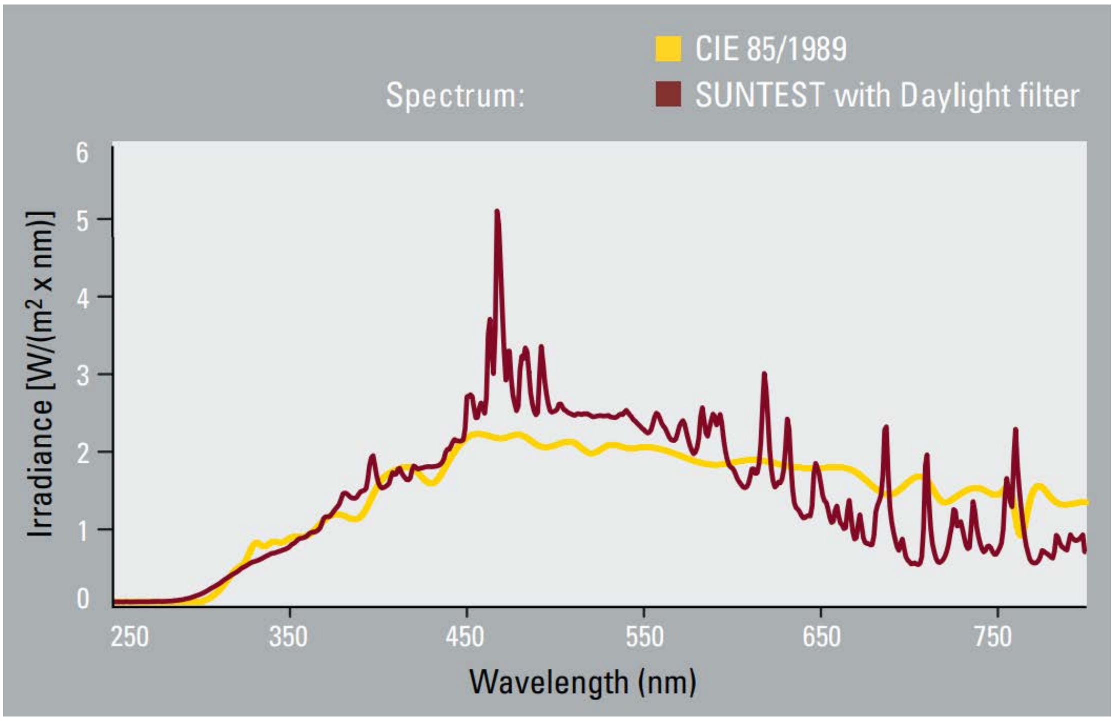

Figure S1. Emission spectrum of the lamp used at the Atlas Suntest CPS+ (Germany) photoreactor.

Table S1. Photocatalytic rate constants (k) and the inhibition percentage Δk% based on the addition of scavengers in the three optimum AOPs.

**Scavengers**	**Photocatalysis** TiO_2		**Photocatalysis MOF-Persulfate**		**Photo-Fenton like** Fe^{2+}**-persulfate**	
	**k (min^{-1})**	**Δk (%)**	**k (min^{-1})**	**Δk (%)**	**k (min^{-1})**	**Δk (%)**
**No scavenger**	5.204	-	0.325	-	1.953	-
**KI**	0.258	95.04	0.015	95.38	-	-
C_3H_8O	0.159	96.95	0.022	93.23	0.035	98.21
NaN_3	2.256	56.65	0.160	50.77	1.118	42.76
